# Digital vertigo therapy: study protocol for a confirmatory randomized controlled trial in patients with vestibular vertigo

**DOI:** 10.1186/s13063-025-08775-0

**Published:** 2025-06-02

**Authors:** Markus Wirth, Jannik Pieper, Ulrike Heller, Michael Bulitta, Daniel Schmitz, Barbara Wollenberg, Hubert Löwenheim, Stephan Wolpert

**Affiliations:** 1 Department of Otolaryngology, RWTH University Aachen, Aachen, Germany; 2 Department of Otolaryngology, Technical University of Munich, Munich, Germany; 3 Chair of Economic and Social Policy, Otto Beisheim School of Management, Koblenz-Vallendar, Germany; 4 Tübingen Study Center, Clinic of Otolaryngology, Tübingen, Germany; 5 CRM Biometrics, Bornheim, Germany; 6 Department of Otolaryngology, University of Tübingen, Tübingen, Germany

**Keywords:** Digital health, Physiotherapy, Vestibulocochlear nerve diseases, DiGA, Digital health application

## Abstract

**Background:**

Vestibular vertigo is one of the leading causes of disability. The clinical standard of care for vestibular vertigo includes physical activity producing central vestibular compensation (CVC). Home exercises are considered an integral part of physical therapy. However, a reliable solution is still needed to support the regular and correct execution of home exercises. For this purpose, VH-90-D DiGA was developed, which is a digital therapeutic (DTx) for multimodular in-home therapeutic training.

**Objective:**

The purpose of this study is to assess the clinical efficacy and safety of a vestibular health app for patients with vestibular vertigo.

**Methods:**

A randomized group-controlled single-blinded clinical trial (RCT) has been designed. Patients will be randomly assigned to one of two treatment groups and the endpoints examined in a pre-determined order. The experimental group receives the DTx (around 15 min/daily for 90 days), and the control receives physiotherapy according to the German statutory health care plan (usually 6 × 20 min of live physiotherapy). The primary outcome will be vertigo intensity measured using the German version of the validated Vertigo Symptom Scale-short form VSS-sf-VER (0–32 score points). Evaluation is performed after 2, 6, and 12 weeks. Primary outcomes are determined by measuring the group differences of the VSS-sf-score point changes from baseline to week twelve. Including dropouts, the sample size has been determined to be 2 × 100.

**Expected results:**

It is expected that therapy with the DTx will be statistically superior to physiotherapy in terms of effect size.

**Discussion:**

This trial protocol marks a confirmatory RCT (GEVE II) to investigate the efficacy and safety of a digital vertigo treatment. The planned RCT is based on a series of primary and secondary efficacy variables. Examination of the endpoints in a pre-determined order ensures the rigor of confirmatory statistics and addresses the challenge of multiplicity. This sequential testing continues until significance is achieved. However, if a specific variable fails to reach significance, subsequent variables will be explored solely on a descriptive basis.

**Trial registration:**

German National Registry of Clinical Studies (DRKS00028026), a WHO ICRTP registry. Registered on December 12, 2023.

## Introduction

### Background and rationale

Vestibular vertigo is common, particularly as non-specific vertigo, and is one of the leading contributors to disability years. Dizziness and vertigo are common causes of medical consultation of general practitioners [1, 2] with neurologists and otolaryngologists. In adults, the lifetime prevalence of any type of dizziness is estimated to be 23.2% [1, 3]. The 1-month prevalence of dizziness varies from 15.8% [4] to 23% [5], and a 1-month prevalence of 10.9% has been reported for dizziness severe enough to interfere with normal activities [5]. The reported 1-year prevalence of vertigo is 48% for adults [6]. Thus, due to it’s high incidence vertigo is a global health problem.

The routine care for vestibular vertigo depends on the underlying diagnosis and ranges from physical therapy and medication to interventional therapies. Physical therapy inducing central vertigo compensation (CVC) is a cornerstone of therapy [7–9] and often an evidence-based drug therapy is not available. In the acute setting, antivertiginous drugs are however regularly prescribed [7]. Antivertiginous drugs can inhibit central vertigo compensation and should therefore be limited in its long-term use[7]. Interventional therapies are rarely performed such as intratympanic administration of cortisone in Meniere’s disease.

Physical therapy is usually performed with a physiotherapist. In addition, home exercises are considered an integral part of physical therapy [10, 11] because long-term interventions are required to activate CVC due to it being based on a central learning process [12]. Consequently, both the evidence-based efficacy of clinical exercise and long-term adherence to the home exercises are critical factors for success [10]. However, a reliable solution is still needed to support regular and correct execution of home exercises and encourage long-term adherence [9, 11, 13–15]. To overcome this problem, a vestibular health app (VH) with 90 application sessions was developed. As a result the unique characteristic of digital therapy is the much higher frequency of therapy sessions in comparison to conventional physiotherapy. The digital vertigo therapy for in-home therapeutic training consists predominantly of adaptive balance movements, eye movements, and visual stimulation (ABEV) exercises; health education; and cognitive behavioral therapy (CBT) interventions together with progressive muscle relaxation (PMS) according to Jacobson, muscle strengthening (Otago program), and autogenous training (AT). Neurophysiological foundation of the therapy provided by the digital therapeutic (DTx) is cerebral vertigo compensation (CVC). CVC is induced by sensomotor and optokinetic exercises provided by the DTx. The sensomotor and optokinetic exercises result in a non-invasive brain stimulation creating the CVC. Users watch and follow video demonstrations and education and read instructions and information. The program is used on smartphones and tablets. A recent systematic review by Grillo et al. 2024 [16] assessed the effectiveness of telerehabilitation. In seven articles significant improvements were observed in the frequency and severity of dizziness, disability, and anxiety, all favoring telerehabilitation.

### Objective

The study objective is to assess the clinical efficacy and safety of VH-90-D DiGA (DTx) for patients with vestibular vertigo.

Two hypotheses are proposed. First, DTx efficacy is hypothesized to show superiority compared to TAU. Attainment of this response would support the second hypothesis that the study can distinguish DTx efficacy from TAU.

### Trial design

This project is a prospective, randomized, group-controlled, single-blinded confirmatory clinical trial (RCT).

## Methods

### Study setting and design

**Fig. 1:**
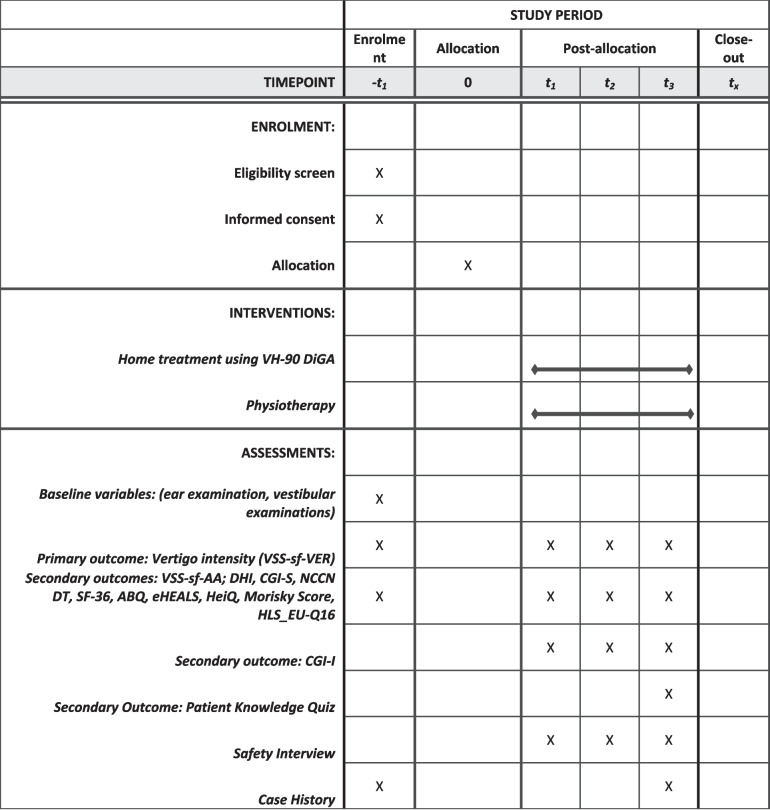
Schedule of enrolment, interventions and assessments (SPIRIT)

This project is a prospective, randomized, group-controlled, single-blinded confirmatory clinical multi-center trial (German Vertigo Study, GEVE II study). Approved by the Ethics Committee of the German State of Baden-Württemberg Chamber of Physicians and Surgeons (F 2021–157) the study has been registered with the German National Registry of Clinical Studies (DRKS00028026). After oral information by a study physician, patients provide written informed consent to participate. In addition to the overall analysis, a separate analysis is planned for the results of each individual center. The scheme in Fig. 1 shows the overall design and clinical procedures of the study. During the follow-up, data acquisition will be performed. After 12 weeks, the study will be complete for an individual patient. If a patient is symptom-free prior to the completion of the study, he/she may discontinue therapy. These patients are not dropouts. After the last patient is out, a complete ITT (intention to treat) analysis will be performed. At T0, study physicians perform the initial clinical assessment in person and submit the results into the uniform online eCRF (electronic case report form, Magana Trial Manager, MaganaMed, Regensburg, Germany). All subsequent data are collected as ePROs using different sections of the same eCRF system. At the designated time points participants receive email links to the questionnaires, along with reminders.

SPIRIT reporting guidelines were used and a SPIRIT checklist is provided in the Supplementary Material [17].

### Study population, recruitment and eligibility criteria

For patient recruitment, doctors at hospitals and private practices will be informed about the study. In addition, newspaper and internet advertisements will be placed to draw attention to the clinical trial. Screening will be based on inclusion and exclusion criteria. A considerable number of patients are expected to fulfill the criteria but are not willing to give informed consent. All participants are investigated and diagnosed by the study physicians who participate in the study as investigators.

Inclusion criteria are age ≥ 18 years and vertigo originating from an ear or vestibular nerve disease. These include the diagnoses in Table 1.

**Table 1:** Selection criteria table

Vertigo diagnoses
Benign paroxysmal positional nystagmus (BPPN), positional vertigo Trauma (e.g., labyrinthine concussion, skull base injuries) Labyrinthitis (toxic, bacterial, viral) Functional vertigo, persistent postural-perceptual dizziness (PPPD), phobic vertigo, chronic vestibular syndrome Tumors of the skull base and cerebellopontine angle, acoustic neuroma Age-related vertigo, presbyvertigo Morbus Menière, Lermoyez syndrome Vestibular migraine Labyrinthine dehiscence Acute vestibular syndrome Vestibular neuronitis Vestibular paroxysmia (neurovascular compression syndrome of the 8th cranial nerve) Bilateral vestibulopathy (bilateral hyperexcitability, bilateral vestibular failure) Unilateral vestibulopathy (unilateral hyperexcitability, unilateral vestibular loss) Hearing loss with vertigo (acute idiopathic sensorineural hearing loss with vestibular involvement) Toxic vestibulopathy (drug side effect, toxic chemicals) Idiopathic vertigo
Exclusion criteria (selected)
Central vertigo/dizziness Severe psychiatric disease Severe neurological disease Cardiovascular vertigo/dizziness Chronic use of antivertiginous drugs Physiotherapy in the 4 weeks before study entry Use of neuroleptics Pregnancy Drug or alcohol abuse Metastases of malignant tumors Acute malignant disease

The investigators assess the inclusion and exclusion criteria through interviews, the evaluation of the VSS-sf-VER questionnaire, and an analysis of technical investigations.

### Informed consent

Patient information, including data protection information and the declaration form for informed consent, will be provided by the physician investigator. After oral clarification by the physician, the patient may voluntarily sign the informed consent. Patients will also be informed that participation is voluntary, explicitly regarding revocability.

### Additional consent provisions for collection and use of participant data and biological specimens

N/A, no ancillary studies.

## Interventions

### Explanation for the choice of comparators

The control group (control group [CG]) will undergo TAU, which is physiotherapy. No therapy cannot be justified, because patients may need therapy. Control patients will receive a physiotherapy prescription and make appointments with a physiotherapist of their choice on their own. No influence will be exerted on timing and possible waiting time, so that these correspond to the TAU procedure.

### Study interventions

The experimental arm (experimental group [EG]) will receive VH-90-D DiGA, Version 1.0.2 D (Digitale Gesundheitanwendung [Digital Health App], legal term according to the German Digital Care Law DVG [2019]. VH-90-D DiGA (Vertidisan®) is a Class I (MDR [18] Medical Device (UDI-DI 42700027834VH90-DN6) is Security Class B standalone software. The software is applied as a digital application to be used on smartphones or tablets. It is manufactured and provided by the manufacturer Digitineers GmbH & Co KG, Tübingen, Germany. Users tap on the device screen to control the application. They watch videos of exercise demonstrations by therapists and instructions by physicians and read instructions and information. The medical contents of the app are based on guidelines [19, 20], expert publications, established handbooks [21, 22], comparable recognized sources [8, 9, 23], or published studies [8, 9, 23–29].

Patients will be asked to use the app daily using the therapy plan provided in the DTx. Daily time consumption for app use is expected to be 10–25 min. During onboarding, a patient may indicate his/her diagnoses. According to the diagnosis, DTx provides 90 therapy days. Experimental patients are free to decide when and where to use the app. Thus, experimental patients choose their individually preferred application time.

The control group (control group [CG]) will receive physiotherapy. Control patients will receive a physiotherapy prescription and make appointments with a physiotherapist of their choice on their own. The physiotherapy consisted of the standard exercise therapy provided for dizziness by physiotherapists licensed to provide care for people with statutory health insurance in Germany and will not get further instructions regarding the trial.

According to the German “Heilmittelkatalog” (catalog of remedies), 6 sessions of 20 min each are possible per prescription, and up to 18 units are recommended as an indicative treatment volume.

To minimize stratification bias, block randomization will be performed. A list of blocks will be created by researchers who are not involved in the performance of the clinical study. Allocation concealment will be achieved by envelope concealment as described by Doig and Simpson [30]. As additional security measures, the person creating the envelope will sign the back of the envelope when sealed. The envelope will be opened no earlier than at the end of the T0 visit to allow patients in the IG to be introduced into the app. Randomization will be performed at the end of the T0 visit by an independent research professional not involved in the trial.

Permanent patient blinding is not feasible. Patients will be blinded during the complete enrollment process until the randomization envelope is opened and patients do or do not receive the app. The researchers collecting the clinical study endpoints will be blinded throughout the study. For ethical and practical reasons, a separate external adverse event (AE) monitoring and app dysfunction committee will be established whose members will be assigned to AE patient care, hazard coping, and app support. In case of a serious adverse event, the German National Authority BfArM is informed indicating expectedness, seriousness, severity, and causality. They will not be blinded and will not collect endpoints. Except for medical emergencies, they will not have contact with patients’ prior endpoints retrieved by the blinded staff. They will contact patients in separate sessions.

### Criteria for discontinuing or modifying allocated interventions

In case of a serious AR, the study will be suspended and data submitted to the ethics committee. In case of a favorable opinion by the committee, the study will be continued.

### Strategies to improve adherence to interventions

Program adherence will be determined by the number of exercise days actually performed. Because adherence to the study is observed by the app, dropouts will be recorded. Treatment compliance will be inferred from the adherence to the DTx program.

### Relevant concomitant care permitted or prohibited during trial

With the exception of the exclusion criteria, other accompanying therapy will be allowed and not influenced by the therapy with the app. Rescue therapy is indicated in the event of life-threatening progression of the underlying disease or a severe deterioration in quality of life. At the end of the clinical trial, the treatment of a patient will not necessarily be complete in all cases. In the IG, the patient can continue to use the app for up to 12 months. In the CG, the use of the app will be available to every patient free of charge after 12 weeks and up to 12 months. Arrangements will be made for additional health care for subjects who suffer from an AE.

### Provisions for post-trial care

Any AE will be recorded during the ongoing study, and measures will be taken by a physician to organize necessary medical care for study participants if such care is required. An external data monitoring committee will be established whose members are responsible for patient care in case of AEs, hazards, or device dysfunction.

### Adverse event and harms reporting

Using the checklist in Table 2, active AE surveillance will be performed. The list results from the extensive literature and database searches in the Vertidisan® Clinical Evaluation Report according to the EU legislation (MDR, Medical Device Regulation, 2017). In addition, a free text option to indicate further complaints will be available.

**Table 2:** Diagnostic procedures

Ear microscopy Spontaneous nystagmus Examination for skew deviation/vertical divergence of the eyes using alternating cover test Examination for peripheral vestibular spontaneous versus central nystagmus with the aid of Frenzel glasses Examination for gaze direction nystagmus in the opposite direction of a possible spontaneous nystagmus or vertical gaze direction nystagmus Head impulse test Romberg test Unterberger test Head shake test Position and positioning nystagmus Caloric testing Objective Standard Balance Deficit Test (SBDT)* Sensory Organization Test (SOT)**	

*The SBDT (Standard Balance Deficit Test) was originally indicated by Allum and Shephard in 1999 [31–33]. Patients perform pre-defined stance/gait tasks. The test includes gyrometric free-field body sway analysis to determine the objective fall risk. Trunk sway is measured by gyrometers at the hip while the subjects carry out the test. Body sway is recorded in the roll (lateral) and pitch (antero-posterior) planes, respectively, at the center of body mass. The SBDT is scored between zero (maximum stability) and 100 (maximum instability)

**The SOT (Sensory Organization Test) was brought forward by Allum et al. in 2002 [35] and by Basta et al. in 2005 [32]. The SOT consists of six conditions to be applied during posturography using a dynamic platform. Posturography is used to determine the objective fall risk. The SOT composite score is between zero (fall) and 100 (maximum stability) [34, 36, 37]

### Outcome measures

#### Baseline data collection

At time point T0, the investigator will examine each patient, take the case history, screen for inclusion and exclusion criteria, and provide oral study information. In case of consent, the patient will provide written informed consent. Eligible participants will be asked to provide information on socio-demographic characteristics, including age, gender, sex, education level, and living situation. Clinical variables, including the presence of any concurrent medical conditions, will also be documented. Participants will also be requested to complete outcome questionnaires. To establish diagnoses, the specific examinations in Table 3 will be performed.

In both tests, a “fall risk” is further diagnosed if the participants move their feet or touch the wall/safety railing during testing [34].

A fall is defined as an unexpected event in which the person inadvertently drops to the ground or another lower level (e.g., stool, bed).

**Table 3:**
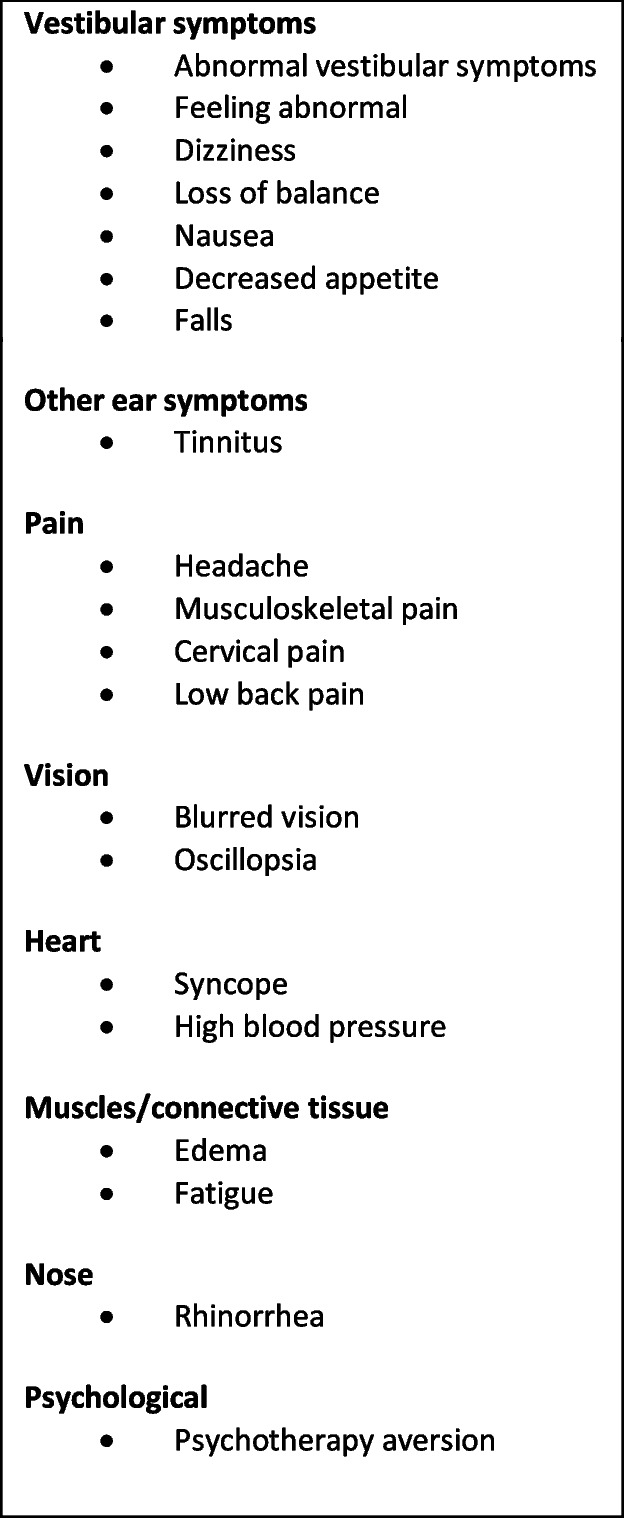
Active surveillance check list for adverse events

#### Primary outcome measure

The primary outcome will be vertigo intensity. This will be measured using the German version of the validated Vertigo Symptom Subscale VSS-sf-VER, which is the vestibular-balance subscale of the German version of the validated Vertigo Symptom Scale (VSS-G) [38, 39]. The instrument measures the frequency of eight vestibular symptoms on a scale from 0 (no symptoms) to 4 (symptoms most days) during the past month (scores 0–32 points). The validity and reliability values are summarized [38–40]:

Reliability: VSS-G: *α* = 0.904 and ICC (CI) = 0.926 (0.826, 0.965).

Discriminant validity: VSS-sf-VER differentiates patients and controls ROC (CI) = 0.99 (0.98, 1.00).

Convergent validity: VSS-G correlates with DHI (*r* = 0.554) and frequency (*T* = 0.317).

#### Secondary outcome measures

*DHI (*Dizziness Handicap Inventory)*, CGI-I* (Clinical Global Impression I – improvement scale), *CGI-S (Clinical Global Impression – Severity Scale),* NCCN® DT (National Comprehensive Cancer Network Distress Thermometer), VSS-sf-AA, SF-36 (36-Item Short Form Health Survey).

Like the primary outcome measure, secondary outcomes will also be evaluated by the authors.

Dizziness Handicap Inventory (DHI)/vertigo handicap.

The Dizziness Handicap Inventory (DHI) is a 25-item, self-reported questionnaire that evaluates the impact of dizziness/vertigo on quality of life and can be used to assess the severity and effect of therapeutic treatments. Each item is rated on a 3-point scale. A higher total score reflects a more severe handicap. The DHI comprises items on three subscales: emotional (DHI-E: anxiety or mental stress influenced by dizziness or vertigo), functional (DHI-F: disability affecting daily living caused by dizziness or vertigo), and physical (DHI-P: dizziness or vertigo provoked by specific self-motions) [31–39, 41–45]. The reliability and validity of the German version have been demonstrated [46]. Cronbach alpha values for the DHI-G and the functional, physical, and emotional subscales were 0.90, 0.80, 0.71, and 0.82, respectively. The limits of agreement were 12.4 points for the total scale (maximum 100 points). Intraclass correlation coefficients ranged from 0.90 to 0.95.

CGI-I/change in vertigo.

The Global Index of Improvement (CGI-I) on the Clinical Global Impression (CGI) questionnaire is a tool to record disease improvement in terms of disease severity and response to therapy [41–45, 47, 48]. The patient's present overall clinical condition is compared to the 1-week period just prior to initiation of the intervention (baseline visit). The query is rated on a 7-point scale: 1 = very much improved since the initiation of treatment; 2 = much improved; 3 = minimally improved; 4 = no change from baseline (the initiation of treatment); 5 = minimally worse; 6 = much worse; 7 = very much worse since the initiation of treatment.

CGI-S/vertigo severity.

The Global Index of Severity (CGI-S) is the second tool of the CGI questionnaire, recording disease severity and response to therapy [43]. The CGI-S is based on the following 7-point scale: 1 = normal, not at all ill; 2 = borderline mentally ill; 3 = mildly ill; 4 = moderately ill; 5 = markedly ill; 6 = severely ill; 7 = among the most extremely ill patients.

NCCN DT/somatic disease-related psychological stress.

The distress thermometer (DT) is a single-item, visual analog scale that can be immediately interpreted to rule out elevated levels of distress [49–54]. The DT measures distress levels over the past week using a thermometer-like Likert scale with scores from 0 (no distress) to 10 (extreme distress) and a midpoint anchor labeled ‘moderate distress’.

VSS-sf-AA/vertigo autonomic responses-anxiety.

Vertigo autonomic responses-anxiety is measured using the German version of the validated Vertigo Symptom Subscale VSS-sf-AA of the VSS-G. The instrument measures the frequency of seven autonomic responses and anxiety symptoms during the past month on a scale from 0 (no symptoms) to 4 (symptoms most days) (total range 0–35 points) [38, 39]. Those may be vestibular-related. Improvement can reflect either fewer or less frequent symptoms.

SF-36/quality of life.

To measure quality of life (QoL) in addition to the DHI, patients will also complete the German version of the generic 36-Item Short Form Health Survey (SF-36) [55, 56].

#### Descriptive endpoints

ABQ (Adherence Barriers Questionnaire).

The ABQ questionnaire is a scale for measuring therapy adherence or the reasons for non-adherence [57].

Hei-Q.

The Hei-Q is an instrument to determine the patient's self-management ability [58]. Only the subscore "acquisition of skills and action strategies" will be examined.

eHEALS.

The eHealth Literacy Scale measures health competence [59]. The eHEALS is an 8-item measure of eHealth literacy developed to measure patients’ combined knowledge, comfort, and perceived skills at finding, evaluating, and applying digital (mostly web-based) health information to health problems. The objective is to psychometrically evaluate gaps in health literacy.

Morisky score.

The theory underlying the 8-item Morisky [57] therapy (originally medication therapy) Adherence Scale (MMAS-8) was that failure to adhere to therapy could occur because of several factors. Response categories are yes/no for each item with a dichotomous response and a 5-point Likert scale for the last item.

##### HLS-EU-Q16

The European Health Literacy Survey questionnaire describes the ability of citizens to make decisions that are beneficial to their health. The higher the health literacy of the individual, the greater the freedom to make decisions in health matters and the greater the ability to locate, understand, and implement health information. The 16 questions can be answered using a pre-determined 4-point response scale (very easy, fairly easy, fairly difficult, very difficult).

Health economics.

The primary methods for evaluating health economics will include cost-effectiveness analysis (CEA) and cost-utility analysis (CUA). This includes determining the expenses associated with all resources (e.g., personnel resources, including physical therapists and physicians, consumables, overhead) consumed as a patient progresses through a care pathway. This comprises waiting times, length of stay, and transportation. The incremental cost-effectiveness ratio (ICER) will be calculated using the effectiveness of app-based therapy and TAU derived from the primary and secondary endpoints. These results will help rank the digital health app in terms of the cost and effectiveness of vertigo treatment. To calculate the costs of all resources, we will employ time-driven activity-based costing (TDABC), a method that combines process mapping and resource-level costing and has been employed globally to assess healthcare costs.

### Follow-up measures

Follow-up measures will be performed after 2 weeks (T1), 6 weeks (T2), and 12 weeks (T3). Questionnaires will be provided digitally. Patients will input their data into the relevant part of the scales or questionnaires. Blinded investigators will input their data into the CRF. Data quality and compliance will be checked by the monitors. To limit loss to follow-up, we will validate with participants’ contact information at each time point. At T1 and T2, the investigators will check with the participants telemedically. Table 4 and 5 provides information on the frequency and timing of clinical measurements.

**Table 4:**
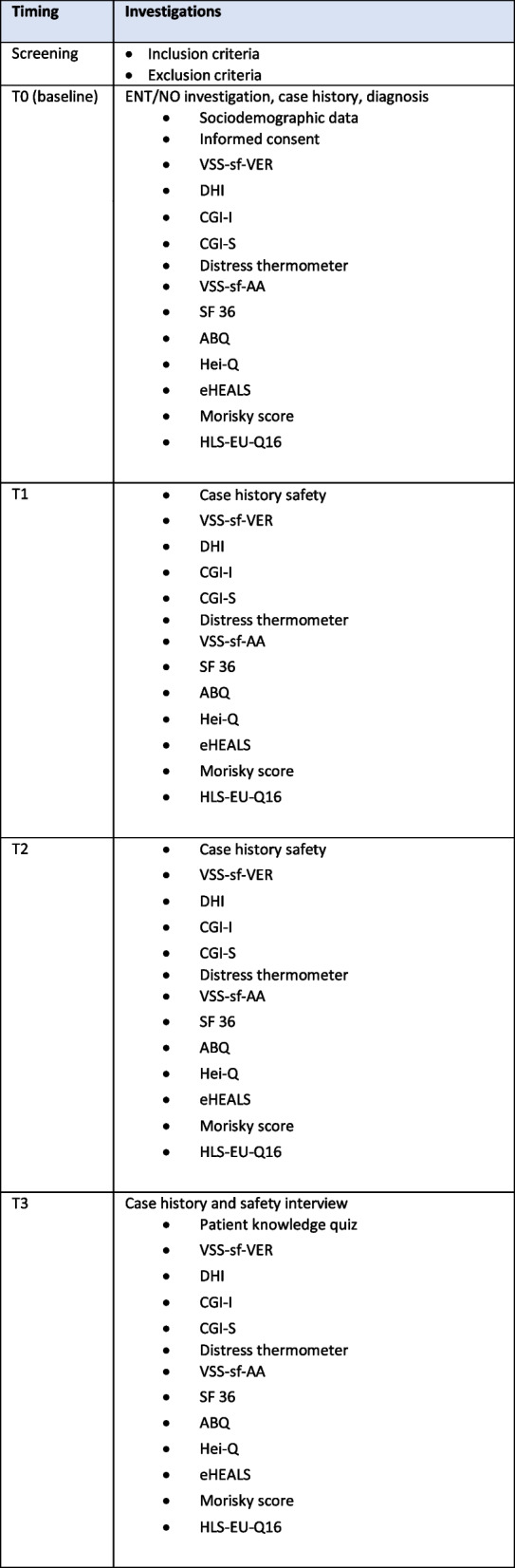
Frequency and timing of measurements

### Participant timeline

**Fig. 2:**
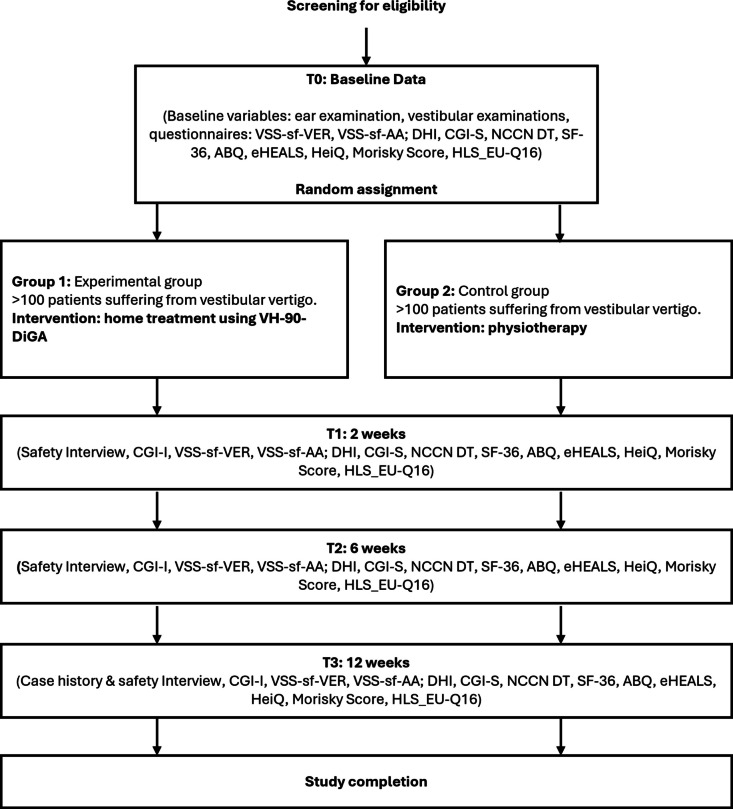
Participant timeline

The schedule of enrolment, interventions, and assessments based on SPIRIT [17] is depicted in Fig. 1. The subsequent scheme shows the overall design and clinical procedures of the study. Major steps are screening, enrollment, allocation, follow-up, and ITT analysis. After screening, the decision on inclusion and/or exclusion is made. The next step is the random assignment and thus the allocation to the experimental group or control group. Within the follow-up, data acquisitions are performed. After 12 weeks the study is completed for each individual patient (Fig. 2).

**Table 5:**
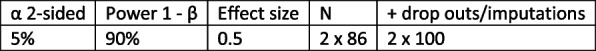
Proposed sample size

### Sample size

The proposed sample size is 200, with 100 per group. The primary endpoint (VSS-sf-VER (German version of VSS-G) has a range of 32. Because no empirical data are available, 15% (IQWiG 2022) [59] of the range was chosen as a relevant effect, i.e., 5; the imputation methods are conservative, and the real effect has to have 6. The standard deviation of the change from baseline to week 12 is expected to be less than 12. To account for the reducing effect of replacing missing values or dropouts, a drop outs/imputations-addition is included, resulting in the final case number calculation.

### Data collection and management

#### Plans for assessment and collection of outcomes

Data will be gathered utilizing a digitized CRF. Outcome measurements will be obtained at the time of inclusion and at follow-up points spanning 2 weeks, 6 weeks, and 12 weeks (Fig. 2).

### Plans to promote participant retention and complete follow-up

To minimize the risk of participants being lost to follow-up, we will periodically verify their contact details and preferred mode of communication at each time point. The study database will be maintained at the Tübingen Study Center (TSC), which will securely store all data collected by the CRF on its own servers. All digitized data, including the Trial Master File and patient identification list, will be consolidated into a single file, encrypted, and archived in the TSC clinical information system. The procedure follows the harmonized Standard Operating Procedures (SOPs) of the German Coordinating Centers for Clinical Studies. Data protection follows the European General Data Protection Regulation.

### Data review, database cleaning and issuing and resolving data queries

Deployment of monitors.

### Verification, validation and securing of electronic clinical data systems

After patients and doctors provide data, it will be viewed by a study monitor. Any discrepancies will be identified and addressed directly with the patient or doctor to avoid incorrect data entry based on misunderstandings or errors.

### Specified retention period

All original data will be stored for the longest possible period of time the TSC permits; at least 15 years according to § 26 AMG.

### Confidentiality

At the conclusion of the study, data will be securely stored in the TSC servers in a de-identified format, identified solely by a unique identification code. Only the investigators will possess access to the key that associates these codes with individuals. The protocol, anonymous data, and statistical code supporting the findings of this study will be available from the corresponding author upon reasonable request.

### Plans for collection, laboratory evaluation and storage of biological specimens for genetic or molecular analysis in this trial/future use

N/A. No biological specimens are collected.

### Statistical methods

For descriptive statistics, usual methods will be used for quantitative variables (i.e., means, medians, standard deviations, quartiles, maximum and minimum). For qualitative variables, absolute and relative frequencies will be determined. Furthermore, 95% confidence intervals (CIs) will be calculated where appropriate. Analysis of covariance (ANCOVA) will be used for the analysis of changes from baseline with baseline as a covariate and treatment group as a factor. No formal pre-tests of normality or homoscedasticity are planned; the range of the score data (changes from baseline) is limited without extreme skewness; the homoscedasticity of the 2 groups of changes is given under the assumption of no treatment effect. The sample sizes of about 100 per group make this simple ANCOVA with 1 factor (group) and 1 covariate (baseline) asymptotically robust even in the case of not normally distributed data. Wilcoxon tests will be used for sensitivity analyses. For confirmatory statistics, the following a priori-ordered tests will be used:

#### Primary confirmatory endpoint

Group comparison of the change in VSS-sf-VER from baseline to Week 12. The primary hypothesis to be tested is the equality of the VSS-sf-VER change against the alternative of the superiority of VH-90-D vs. TAU with a two-sided significance level of α = 5%. ANCOVA with experimental group as factor and baseline as covariate and sensitivity analysis with a Wilcoxon rank test stratified by baseline values will also be carried out.

### Secondary confirmatory endpoints

Group comparison of DHI change T0-T3 (ANCOVA), group comparison of CGI-I at week 12 (Wilcoxon-test), group comparison of CGI-S changes T0-T3 (ANCOVA), group comparison of DT changes T0-T3 (ANCOVA), group comparison of VSS-sf-AA changes T0-T3 (ANCOVA), group comparison of SF-36 changes T0-T3 (ANCOVA).

All tests of secondary endpoints (#2–7 above) will be 2-sided tests with *α* = 5%. For handling multiplicity, including alpha error avoidance, the above-listed endpoints will be tested in the above indicated a priori order as long as significance is reached; if significance is not reached for a certain variable, further listed variables can be tested only in a descriptive manner. This a priori rejecting procedure controls the multiple significance level of 5% in a strong sense for all primary and secondary endpoints.

All post hoc tests including subgroup analyses are planned as descriptive statistics with exploratory interpretation of p values and 95% CIs.

#### Internal validity

The randomization sequence will be established before the study participants are assigned to the different groups. At the time of study inclusion by the study physician, the randomization will still be secret. Thus, the selective selection of study participants into the different treatment groups is sufficiently counteracted.

Due to the lack of blinding of the patients to participation in the app intervention or physiotherapy, performance bias will be present to a certain degree, which has to be accepted due to the impracticable blinding. In the self-reported endpoints (patient-reported outcomes), there is no detection or observer bias. However, the functionality and AE surveys by separate, non-blinded external committee members are subject to detection bias. This is acceptable in that the functionality and AE survey is descriptive and do not include confirmative endpoints. Reporting bias will be minimized through pre-specification of the primary and secondary confirmative endpoints and statistical procedures including statistical handling of dropouts.

Attrition bias will be minimized by evaluating the ITT population using multiple imputation as a pre-specified procedure. In addition, the reasons for missing data and drop-outs will be collected and evaluated.

#### Subgroup analyses

Subgroup analyses will be performed by age (< = / > 65), disease, disease type (permanent/attacks), disease duration, number of therapy/use days, falls/falling risk, responders, and disease severity/intensity/handicap.

#### Protocol non-adherence and missing data

Participants withdrawing from the study and reasons for withdrawal will be analyzed. Characteristics of participants and non-participants will be compared. Multiple imputation will be used to handle missing data. The primary analysis set is the FAS for ITT analysis. The secondary analysis set is the PP set. Drop-outs will be replaced with reference-based multiple imputation – “Jump to reference” [60]. This method is conservative in handling the attrition bias. Other methods will be applied as sensitivity analyses (e.g., LOCF, BOCF, and total mean replacement). The estimand will be the effect achievable in patients with an affinity for apps vs. TAU. Patients who are symptom-free prior to completion of the study are free to discontinue the therapy. These patients are not dropouts.

#### Plans to give access to the full protocol, participant-level data and statistical code

The protocol, data, and statistical code that support the findings of this study will be available from the corresponding author, upon reasonable request.

### Oversight and Monitoring

#### External data monitoring committee, its role and reporting structure

An external data monitoring committee is established whose members are responsible for patient care in case of AEs, hazards, or device dysfunction.

#### Adverse event reporting and harms

Using the checklist in Table 3, an active AE surveillance is performed. The list results from the extensive literature and data bank searches in the Vertidisan® Clinical Evaluation Report according to the EU legislation (MDR 2017). In addition, a free text option to indicate further complaints is available.

#### Auditing

The sponsor organizes weekly online data monitoring. In case of missing or inconsistent data central auditing is performed by monitors. Furthermore, the sponsor organizes at least one on site audit in each study centet.

## Discussion

In the treatment of chronic vertigo physiotherapy is effective [7–9]. Its application, however, by a physiotherapist is often restricted to a limited number of sessions. Adherence to home-based therapy is frequently insufficient, prompting the development of VH-90-D DTx [9, 11, 13–15]. This study aims to evaluate the DTx´s efficacy and safety. A further focus is on the comparative advantages of digital therapy including benefits for the patient, impact on quality of life, technological developments in rehabilitation, costs, and accessibility. The instruments used allow to investigate vertigo intensity, vertigo-induced loss of quality of life and handicap, disease improvement, vertigo associated stress, adherence barriers, health competence, and health decision-making competence. This trial protocol marks a confirmatory RCT (GEVE II) to investigate the efficacy and safety of DTx for vertigo by VH-90-D DiGA (Vertidisan®). VH-90-D DiGA is a medical device, standalone software, and a health app. DTx allows a multimodal approach and contains vestibular exercises that may be designated as ABEV stimulation exercises. The general effectiveness of vestibular exercises for the treatment of peripheral vertigo has been demonstrated by clinical evidence and is based on cerebral vertigo compensation (CVC) [40]. Recently, an own study of ABEV exercises was completed and served as a foundation for the current trial [61]. In the study, a retrospective analysis of data from a cohort of 104 intra-individually controlled patients demonstrated that vertigo-specific ABEV stimulation exercises are significantly effective in reducing peripheral vestibular vertigo. Cohen's d showed large effect sizes. Thus, the data provide high real-world effectiveness at Oxford Evidence Level 2a and Grade B. From this intra-individually controlled data alone, we can infer statistical probability and clinical effect size but not necessarily the causality of the ABEV stimulation exercises. Furthermore, the published study has some limitations that may affect the internal and external validity of the results. The enrollment followed a consecutive selection mechanism. Moreover, a relevant loss-to-follow-up was noted, which is typical for retrospective studies. The drop-outs may produce bias.

The present RCT was developed to overcome these problems. However, though characterized by a confirmatory design, the experimental nature of the trial may introduce bias. This includes the contact with study physicians. Formally not considered a confounder, it could introduce bias. Exposure to study doctors may contribute to better study adherence in the RCT compared with real-life studies. Furthermore, the absence of blinding for patients regarding the care provided may influence their perceptions. However, this approach is deemed essential because patients experience their specific treatment. Nevertheless, due to the nature of the trial, patient awareness of the specific care model they encounter may enhance external validity.

The planned RCT is based on a series of primary and secondary efficacy variables. Vertigo intensity is measured using the German version of the VSS-sf-VER. The factor structure of the VSS and the validity and reliability of the VSS-sf-VER have been assessed for various languages [38], including German [39, 46]. The DHI uses the original three-subscale structure from various factorial solutions of the DHI [38]. The reliability and validity of the German version has been demonstrated [42]. The CGI has been shown to correlate well with standard, well-known efficacy scales (e.g., Hamilton Rating Scale for Depression, Hamilton Rating Scale for Anxiety, Positive and Negative Syndrome Scale, Leibowitz Social Anxiety Scale, Brief Psychiatric Rating Scale, Scale for the Assessment of Negative Symptoms) across a wide range of indications. Although originally developed for psychiatry, it is now also widely used for somatic diseases, including vestibular disease [46]. The DT has been proven to be feasible, accessible, and informative [49, 53]. Vertigo autonomic responses/anxiety are measured using the German version of the validated VSS-sf-AA. The factor structure of the VSS-sf-AA and its validity and reliability have been assessed for various languages, [38] including German [39]. The SF-36 Health Survey is a widely used classical and validated inventory.

The study is designed as a confirmatory RCT. To ensure the rigor of confirmatory statistics and address the challenge of multiplicity, we will examine the listed endpoints in the pre-determined order outlined in the Methods. This sequential testing will continue until statistical significance is achieved. However, if a specific variable fails to reach significance, subsequent variables will be explored solely on a descriptive basis. This a priori rejection protocol effectively manages the overall significance level at 5% with a robust control mechanism for both the primary and secondary endpoints. Primary outcomes are determined by measuring the group differences of the VSS-sf-score point changes from baseline to week twelve. Even if VSS-sf-score point changes from baseline to week twelve within the experimental group is large, group difference of changes might not be clinically significant.

The expected benefit is sustainable execution of the ABEV exercises and participation in the CBT and health education program, all contributing to reducing vertigo, stress, and fall risk and improving QoL and health knowledge**.** The improvements are anticipated to achieve a clinically meaningful effect size, quantified using Cohen’s d**.** Another benefit may be the reduction of a patient's burden, as they can perform the therapy at home and avoid the burden of travel or transport to physiotherapy sessions. In addition, the waiting time until the start of physiotherapy is avoided.

## Trial status

The first patient in was July, 8th, 2023. The last patient out is expected on May 15, 2025. The actual Protocol version is 6.0 (December 20, 2022). The Protocol was initially submitted to the journal on 8th November 2023 and was re-submitted after the close of the study due to technical problems in the initial submission process.

## Data Availability

The data of the study will be published in a peer reviewed journal.

## Abbreviations

ABEV: Adaptive balance movements, eye movements, and visual stimulation
ABQ: Adherence Barrier Questionnaire
AE: Adverse event
ANCOVA: Analysis of covariance
AR: Adverse reaction
AT: Autogenous training
BfArM: Bundesinstitut für Arzneimittel und Medizinprodukte (German FDA)
BOCF: Baseline Observation Carried Forward
BPPN: Benign paroxysmal positional nystagmus
CBT: Cognitive behavioral therapy
CEA: Cost-effectiveness analysis
CG: Control group
CGI: Clinical Global Impression Index
CGI-I: Clinical Global Impression Index—Improvement
CGI-S: Clinical Global Impression Index—Severity
CI: Confidence interval
CRF: Clinical report form
CUA: Cost-utility analysis
CVC: Central vestibular compensation
DHI: Dizziness Handicap Inventory
DiGA: Digitale Gesundheitanwendung (Digital Health App). Legal term according to the German Digital Care Law DVG (2019)
DRKS: Deutsches Register Klinischer Studien (German clinical study register)
DT: Distress thermometer
DTx: Digital therapeutics
eCRF: Electronic clinical report form
EG: Experimental group
eHEALS: EHealth Literacy Scale
ENT: Ear nose and throat
FAS: Full analysis set
GCP: Good Clinical Practices
GEVE: German Vertigo Study
Hei-Q: Health Education Impact Questionnaire
HLS-EU-Q16: The European Health Literacy Survey questionnaire
ICER: Incremental cost-effectiveness ratio
ICRTP: International Clinical Trials Registry Platform
IG: Interventional group
IQWIG: German Institute for Quality and Efficiency in Healthcare
IRB: Institutional Review Board
ITT: Intention to treat
JTR: Jump to reference
LOCF: Last observation carried forward
MDR EU: Medical device regulation
MMAS-8: Morisky Medication Adherence Scale
NO: Neurootological
PMS: Post Market Surveillance
PPPD: Persistent postural-perceptual dizziness
QoL: Quality of life
RCT: Randomized controlled trial
SAE: Serious adverse event
SAR: Serious adverse reaction
SBDT: Standard Balance Deficit Test
SF 36: Short Form 36 (QoL Questionnaire)
SOP: Standard operating procedure
SOT: Sensory Organization Test
TAU: Treatment as usual
TDABC: Time Driven Activity Based Costing
TSC: Tübingen Study Center
VH-90-D: Vestibular Health App, 90 application sessions, German (D) version
VSS-G: Vertigo Symptom Scale Short Form (German)
VSS-sf-AA: Vertigo Symptom Scale Short Form, autonomic responses-anxiety subscale
VSS-sf-VER: Vertigo Symptom Scale Short Form, vestibular-balance subscale subscale
WHO: World Health Organization
